# Preparation of Tannic Acid-Pectin Coated PVDF Membrane for High-Efficiency Separation of Oil and Water Emulsions

**DOI:** 10.3390/membranes15050155

**Published:** 2025-05-16

**Authors:** Liangku Zhai, Jiuyun Cui, Lei Lu, Hailong Wang, Can Wei, Jirong Luo, Atian Xie

**Affiliations:** 1 School of Materials Science and Engineering, Anhui University of Science and Technology, Huainan 232001, China; 2 Anhui International Joint Research Center for Nano Carbon-Based Materials and Environmental Health, Anhui University of Science and Technology, Huainan 232001, China; 3 Hangmo New Materials Group Co., Ltd., Huzhou 313300, China

**Keywords:** superhydrophilicity, tannic acid, pectin, PVDF membrane, oil–water separation

## Abstract

The simple preparation of superhydrophilic membranes with good stability is of great significance for efficient oil–water separation. In this work, a polyvinylidene fluoride (PVDF) membrane modified with tannic acid (TA) and pectin (PT) was developed through immersion in TA/PT solutions, facilitating the formation of complexes via co-deposition. The optimized PVDF@TA/PT3 membrane exhibited superhydrophilicity/superoleophobicity. The membrane achieved remarkable separation efficiencies exceeding 98.3% and fluxes ranging from 71.3 to 156.3 L m^−2^ h^−1^ for various oil–water emulsions under gravity-driven conditions. Notably, the membrane maintained exceptional durability through 10 separation cycles, retaining about 98% efficiency while exhibiting strong antifouling properties. Excellent separation performance coupled with facile fabrication protocol and chemical stability of the membrane, position the PVDF@TA/PT membrane as a technologically viable candidate for wastewater purification.

## 1. Introduction

Oily wastewater, a by-product of industrial activities including petrochemicals, food processing, textiles, and pesticide manufacturing, poses a significant threat to the natural environment and human health due to its complex composition of emulsified hydrocarbons, toxic organic compounds, and suspended solids [1,2,3]. Common methods for treating oily wastewater include precipitation (limited by sludge generation), centrifugal separation, adsorption (constrained by saturation capacity), membrane separation (versatile for various oil concentrations), and advanced oxidation (effective but costly for large-scale applications) [4]. Among these techniques, membrane separation has garnered significant attention owing to its low energy consumption (operating primarily via pressure-driven mechanisms), straightforward operational requirements (minimal chemical additives), and broad applicability across acidic, alkaline, or high-salinity conditions [5,6,7,8]. However, traditional membranes suffer from inherent limitations: their hydrophobic surfaces readily adsorb oil droplets through interfacial interactions, while membrane pores become obstructed by viscous oil phases or surfactant-stabilized emulsions, collectively causing irreversible fouling and compromised separation accuracy [9,10,11,12]. Therefore, it is urgent to develop oil–water separation membranes with high antifouling properties.

In recent decades, superhydrophilic membranes have attracted considerable interest for oil–water separation due to their ability to promote the formation of hydration layers and repel oil droplets [13,14,15,16,17]. For example, Cheng et al. [18]. fabricated a biodegradable superhydrophilic membrane through electrospinning technology, aiming for ultra-fast purification of oily water. Zeng et al. [19]. developed a light Fenton self-cleaning superhydrophilic membrane through polymerization toward microalgae dehydration and oil–water emulsion separation. Nevertheless, the methods for preparing superhydrophilic membranes are often cumbersome and expensive, which to some extent limits their widespread application [20,21]. Polyphenols, particularly tannic acid (TA), have gained attention due to their environmental friendliness and economic benefits. TA contains numerous hydrophilic groups, making it suitable for hydrophilic modification of membranes [22,23]. For instance, Zuo et al. [24]. prepared gelatin-tannic acid coatings (FOGE-TA) applicable to different substrates by dip coating for oily wastewater treatment. Wang et al. [25]. fabricated a membrane with superhydrophilicity and underwater superoleophobicity using ovalbumin (OVA) and TA for oil–water separation. Xie et al. [26]. produced hydrophilic PVDF membranes through inkjet printing for emulsion separation. While the stability of modified membranes prepared using TA as described above is poor in strong acid and alkali solutions and has certain limitations. The natural polysaccharide pectin (PT), predominantly extracted from renewable biomass sources such as citrus peel and apple pomace, possesses unique molecular characteristics that make it particularly suitable for membrane modification. Its linear galacturonic acid backbone contains abundant free carboxyl groups (-COOH) and hydroxyl (-OH) moieties, which not only confer strong hydration capacity through hydrogen bonding with water molecules but also provide multiple reactive sites for chemical crosslinking. This structural advantage enables PT to serve as an effective hydrophilicity enhancer when incorporated into membrane coatings, simultaneously improving surface wettability and reducing interfacial energy [27,28]. Therefore, it is expected to construct stable superhydrophilic coatings through the co-deposition of PT and TA.

Here, a PVDF@TA/PT membrane with superhydrophilicity was developed using the co-deposition method for the separation of oil–water emulsion. The chemically stable and antioxidant PVDF membrane was used as a substrate, and a TA/PT mixed coating was deposited on the base membrane surface using a facile cyclic deposition method. The binding between TA and PT is achieved through hydrogen bonding, and due to the strong polarity of the N and F elements in commercial PVDF membranes, the PVDF membrane is also bound to TA and PT through hydrogen bonding. The formed TA/PT is rich in hydroxyl and carboxyl groups with strong hydrogen bonding interactions, which not only endows the base membrane with good superhydrophilicity but also exhibits good stability. The prepared PVDF@TA/PT membrane can efficiently separate different kinds of oil–water emulsion. Moreover, the obtained membrane shows environmental stability and maintains its superwettability even in acidic, alkaline, and salt media. The TA/PT co-deposition hydrophilic modification strategy proposed in this work has the advantages of simplicity, safety, and environmental friendliness and has broad research potential in the field of hydrophilic modification of membrane surfaces.

## 2. Materials and Methods

### 2.1. Materials

Polyvinylidene fluoride (PVDF) membrane (Φ = 47 mm) was provided by Haining Filter Equipment Factory. Liquid paraffin, xylene, hexane, petroleum ether, sodium dodecyl sulfate (SDS), kerosene, and anhydrous ethanol were obtained through Sinopharm Chemical Reagent Co., Ltd. (Shanghai, China). Tannic acid (TA) and pectin (PT, galacturonic acid ≥ 74.0%) were derived through Aladdin Biochemical Technology Co., Ltd. (Shanghai, China) Sodium hydroxide (96%), dilute hydrochloric acid (10%), and sodium chloride (99.5%) were obtained through Macklin Biochemical Co., Ltd. (Shanghai, China).

### 2.2. Synthesis of PVDF@TA/PT Membrane

**Figure 1:**
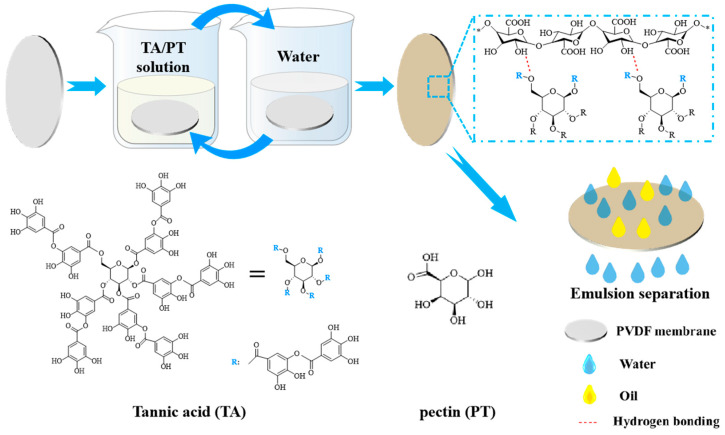
Schematic diagrams PVDF@TA/PT membrane preparation.

Initially, 100 mg of TA and PT were dissolved separately in 50 mL of deionized water to prepare TA solution and PT solution, respectively. These solutions were then mixed and stirred for about 1 h to prepare the TA/PT mixture solution. Subsequently, the PVDF membrane was subjected to a process of wetting with ethanol, followed by immersions in the TA/PT solution and deionized water for 3 min each, constituting a cycle. The membranes were prepared with 1, 3, and 5 cycles, designated as PVDF@TA/PT1, PVDF@TA/PT3, and PVDF@TA/PT5, respectively. Additionally, the membranes were prepared using TA or PT solutions for 3 cycles, labeled as PVDF@TA3 and PVDF@PT3. The fabrication process is shown in Figure 1.

### 2.3. Characterizations

The characterization of the surface structure and element of materials was conducted using a scanning electron microscope (SEM, TESCAN MIRA LMS, Czechia, Brno, Czech Republic) equipped with energy dispersive X-ray spectroscopy (Smartedx, Oberkochen, Germany). The characterizations of membranes were analyzed by an aperture analyzer (CFP-1500AE, Corvallis, OR, USA). The functional groups on the membrane surface were analyzed using Attenuated Total Reflection-Fourier Transform Infrared spectroscopy (ATR-FTIR, Madison, WI, USA), collected over a range of 500–4000 cm^−1^ at 2 cm^−1^ resolution using a Nicolet 560 spectrometer. X-ray photoelectron spectroscopy (XPS, Waltham, MA, USA) was estimated by a Thermo Scientific K-Alpha spectrometer equipped with an Al Ka X-ray source (1486.6 eV); the survey scan range was 0–1360 eV with a step size of 1 eV and a sweep number of 5. Survey spectra were recorded with a pass energy of 150 eV and high-resolution spectra with a pass energy of 50 eV. Water contact angles (WCAs) and underwater oil contact angles (UOCAs) were measured with a contact angle tester (OSA60, LAUDA Scientific, Osnabrück, Germany). Deionized water (5 μL) was carefully added to the membrane surface using a microsyringe, and the WCA between the membrane and water droplets was continuously recorded for 10 s. 1,2-Dichloroethane (5 μL) was dropped onto the surface of the membrane immersed in water using a microsyringe, and the UOCA between the membrane and the oil droplet was recorded after stabilizing for 5 s. Each sample was measured three times in different regions, and the average value was given.

### 2.4. Emulsion Separation Performance

40 mg SDS and 1 mL oil (petroleum ether, liquid paraffin, n-hexane, or kerosene) were dissolved in 99 mL deionized water. The mixtures were stirred for 12 h to obtain milky oil–water emulsions. The separation performance of membranes was evaluated through a filtration test with an operation area of 1.8 cm^2^. The membrane flux *J* (L m^−2^ h^−1^) was evaluated by recording the filtrate volume (1):

(1) $$J=\frac{V}{A\times ∆t}$$

where *V* (L) is the permeate volume, *A* (m^2^) is the effective membrane area, and ∆*t* (h) is the operating time. The alteration in emulsion concentration prior to and following separation was ascertained by means of a UV-visible spectrophotometer. The separation efficiency, *R* (%), was calculated using Equation (2):

(2) $$R=\frac{C_{0}-C_{f}}{C_{0}}\times 100\%$$

where *C_f_* and *C*_0_ denote the oil content of the filtrate and original emulsion.

### 2.5. Stability of Membrane

The various treatments were conducted to assess the stability of the membrane: 60 bending/folding cycles, immersing in deionized water with stirring for 12 h, and immersing in deionized water with ultrasonic treatment for 20 min. In addition, the membrane was soaked in hydrochloric acid solutions with pH values of 1, 3, and 5, as well as sodium hydroxide solutions with pH values of 9, 11, and 13, respectively, for a period of 12 h. Moreover, the membrane was soaked in NaCl solution (0, 1, 2, 3, 4, and 5%) for a duration of 12 h. Finally, the contact angle and separation performance were tested to evaluate the membrane stability.

## 3. Results and Discussions

### 3.1. Characterization of Membranes

**Figure 2:**
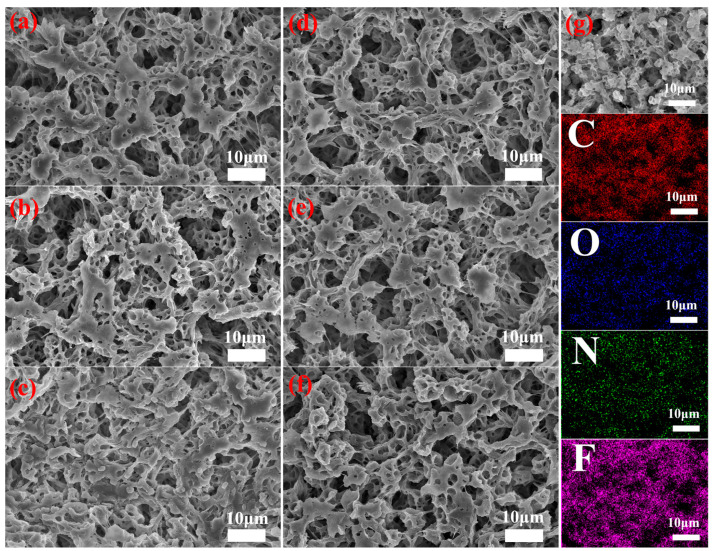
(**a**–**f**) SEM images of as-prepared membranes, (**g**) EDS mapping of PVDF@TA/PT3 membrane.

Surface morphologies of as-prepared membranes are presented in Figure 2. As illustrated in Figure 2a, the pure PVDF displays a porous structure. Additionally, the modified PVDF membrane still maintains obvious porous characteristics, but there is a polymer layer deposited on the surface (Figure 2b–f). The EDS mapping of the elements for PVDF@TA/PT3 membrane (Figure 2g) shows the uniform distribution of C, N, O, and F elements. The results indicate that the deposition of TA/PT on the membrane surface is uniform.

**Figure 3:**
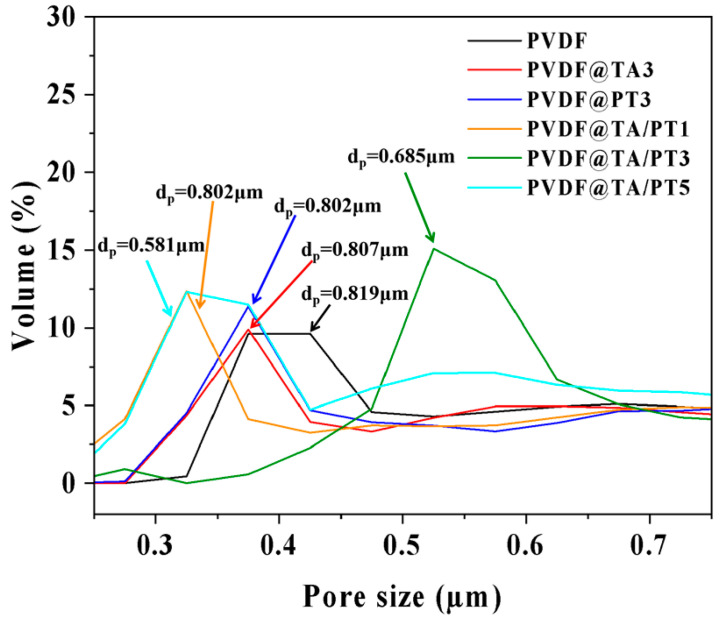
The analysis of the aperture of membranes.

Figure 3 depicted the pore size distribution of as-prepared membranes. The average pore size (d_p_) of the PVDF is 0.819 µm. After the deposition process, the d_p_ of the membranes gradually decreases. The d_p_ of PVDF@TA3 and PVDF@PT3 control membranes are 0.807 µm and 0.802 µm, respectively. After co-depositing PT/TA once, the d_p_ of PVDF@TA/PT1 membrane is 0.802 µm. As the number of depositions increases, the d_p_ of PVDF@TA/PT3 and PVDF@TA/PT5 membranes decreases to 0.685 µm and 0.581 µm, respectively. The results demonstrate the PVDF@TA/PT has been successfully prepared, thereby the retention of sub-micron pores is conducive to the separation of emulsion.

**Figure 4:**
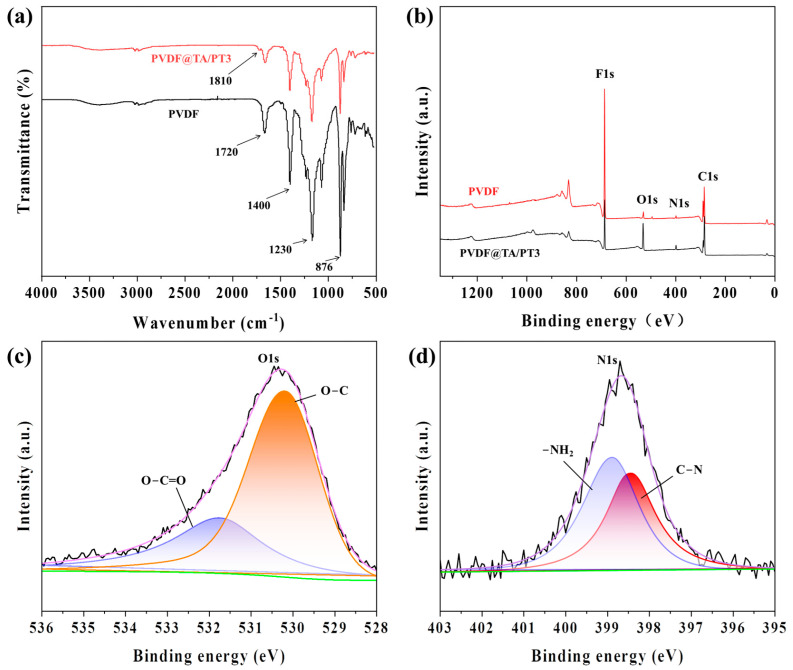
ATR-FTIR (**a**) and XPS (**b**) of PVDF and PVDF@TA/PT3 membranes, and the XPS spectra for O1s (**c**) and N1s (**d**) of the PVDF@TA/PT3 membrane.

The surface chemical composition of the prepared membranes was determined by ATR-FTIR spectroscopy. The ATR-FTIR spectrum of the PVDF membrane reveals characteristic peaks of PVDF at 1720 cm^−1^, 1400 cm^−1^, 1230 cm^−1^ and 876 cm^−1^, corresponding to C=O stretching, –CH_2_ planar mixing vibration, –CF_2_ stretching, and C–C skeleton vibration, respectively [29,30,31]. A new peak at 1810 cm^−1^ in the PVDF@TA/PT3 membrane spectrum is attributed to the C=O stretching vibration of TA and PT, thereby confirming the successful co-deposition onto the membrane surface (Figure 4a). Furthermore, the element composition of PVDF and PVDF@TA/PT3 membranes was characterized by XPS. The XPS analysis results show that the O1s signal in the PVDF@TA/PT3 membrane spectrum is significantly enhanced (Figure 4b). High-resolution XPS spectra for O1s and N1s (Figure 4c,d) distinctly show the presence of O=C–O, O–C, C–N, and –NH_2_ groups [32], corresponding to peaks at 531.76, 530.23, 398.42, and 398.93 eV, respectively, unequivocally indicating the successful coating of TA and PT onto the PVDF membrane. The N element of PVDF is attributable to the purchased commercial PVDF membrane [33]. Concurrently, the presence of –NH_2_ facilitates electrostatic binding with –COOH. Such results were consistent with previous analytical results obtained by EDS and ATR-FTIR spectroscopy.

### 3.2. Surface Wettability

**Figure 5:**
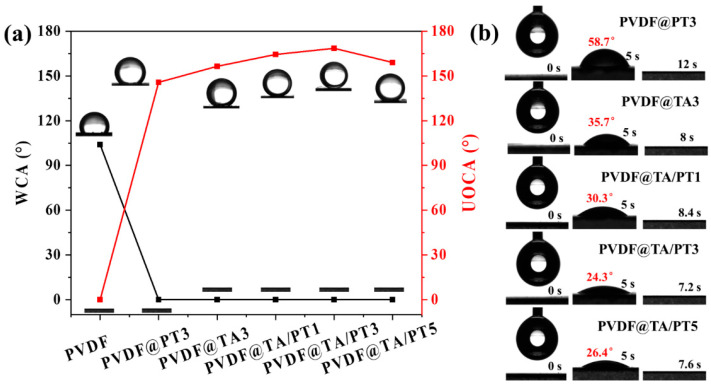
(**a**) WCA and UOCA of membranes under different modification conditions, (**b**) the time-dependent WCA under different membranes.

The wettability of the membrane is an important indicator affecting the oil–water separation ability, and it has been carefully examined. As depicted in Figure 5a, the PVDF membrane exhibits a WCA of 104°, indicating inherent hydrophobicity. In contrast, all modified membranes display WCAs of 0°, signifying TA or PT coating on the membrane surface can greatly improve their hydrophilicity. The UOCA of the PVDF, PVDF@TA3, and PVDF@PT3 membranes was 0°, 156.5°, and 145.8°, respectively. Notably, the PVDF@TA/PT3 membrane boasts a UOCA of 168.6°, suggesting the co-modification of TA and PT on the PVDF membrane surface could further improve its underwater oleophobicity. The dynamic WCA test (Figure 5b) highlights that the WCA of the PVDF@TA/PT3 membrane rapidly transitions to 0° within 7.2 s, indicating its excellent hydrophilicity.

### 3.3. Separation Performance of Membrane

**Figure 6:**
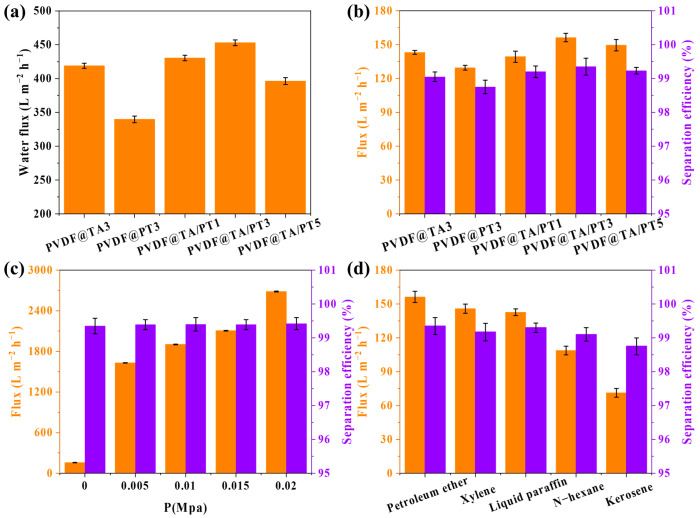
(**a**) Water flux and (**b**) separating property of different membranes; (**c**) separation performance of petroleum ether emulsion at different pressures; and (**d**) separation performance of various emulsions for PVDF@TA/PT3 membrane.

The present study aims to assess the separation property of membranes using petroleum ether-water emulsions by conducting a series of separation experiments. Figure 6a shows the water fluxes of various membranes under gravity-driven conditions, and the PVDF@TA/PT3 membrane has a maximum flux of 452.9 L m^−2^ h^−1^, which is consistent with the wettability results. Figure 6b displays the fluxes of the PVDF@TA/PT1, PVDF@TA/PT3, and PVDF@TA/PT5 membranes as 139.3 L m^−2^ h^−1^, 156.3 L m^−2^ h^−1^, and 149.5 L m^−2^ h^−1^, and corresponding separation efficiencies are 99.2%, 99.4%, and 99.2%, respectively. The PVDF@TA/PT3 membrane showed optimal flux and efficiency of 156.3 L m^−2^ h^−1^ and 99.4%, respectively. The results indicated TA and PT co-deposition on the PVDF membrane surface could further improve its performance. Considering both separation flux and separation efficiency, the subsequent experiments were conducted using the PVDF@TA/PT3 membrane. Figure 6c shows the correlation between pressure and separation performance. The results showed that as the pressure gradually increased to 0.02 MPa, the separation efficiency of the emulsion remained at about 99.3%, while the separation flux increased sharply. Figure 6d shows the separating property of PVDF@TA/PT3 for various emulsions under gravity. The separation efficiency exceeds 98.3%, and the separation flux ranges from 71.3 to 156.3 L m^−2^ h^−1^, indicating the PVDF@TA/PT3 membrane has an outstanding separation effect on various emulsions.

**Figure 7:**
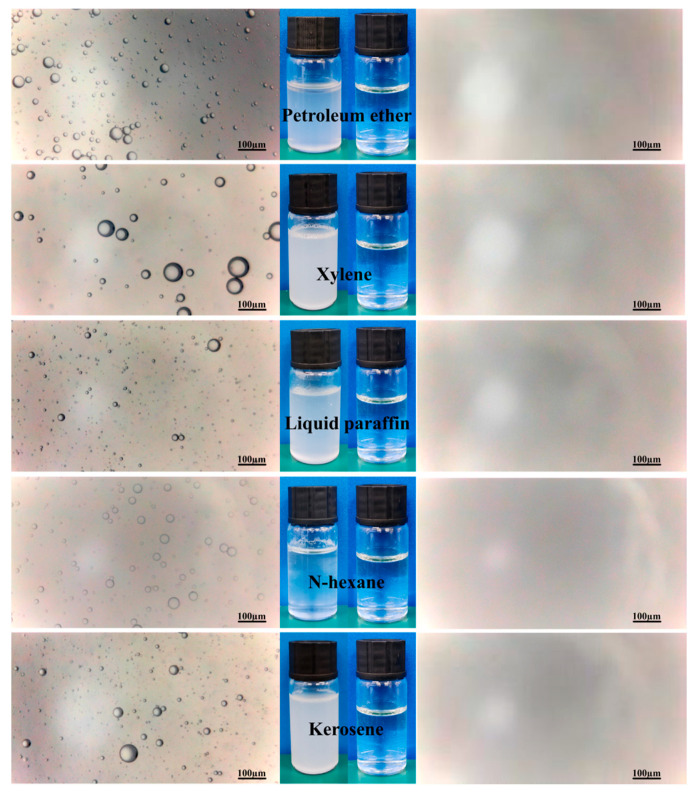
Photos of emulsions (**left**) and filtrate (**right**) and optical microscope images.

Figure 7 shows photographs and optical pictures of various emulsions both before and after separation. Before separation, the emulsions were milky white, and a lot of oil droplets were clearly visible in optical images. After separation, the filtrates all became transparent and colorless, and no oil droplets were observed in the filtrates. The combined action of TA and PT endows the membrane with supperwettability, thus inhibiting the oil droplets’ contact with the membrane directly in separation and effectively intercepting oil droplets, and shows good emulsion separation performance.

### 3.4. Stability of Membranes

**Figure 8:**
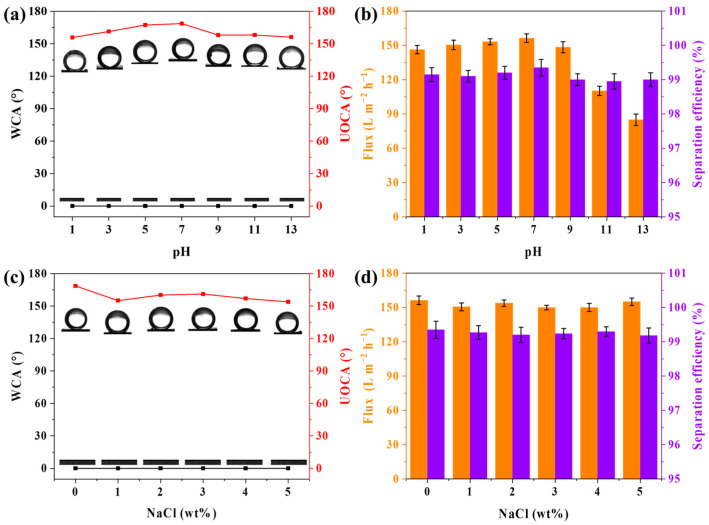
(**a**) WCA and UOCA and (**b**) separation performance of treated membranes at different pH values; (**c**) WCA and UOCA and (**d**) separation performance of treated membranes in salt concentrations that are different.

Figure 8a shows the UOCA and WCA of the membranes treated with solutions with different pH values. The WCAs of the treated membranes are 0°, and UOCAs range from 155.8° to 168.6°. The PVDF@TA/PT3 membrane maintains superhydrophilicity and underwater superoleophobicity in alkaline and acidic environments. As shown in Figure 8b, the PVDF@TA/PT3 membrane showed slightly different separation performance for acid oil–water emulsion, but the separation efficiency remained above 99%. However, the separation flux of the PVDF@TA/PT3 membrane separates in an alkaline oil–water emulsion and changes significantly, which may be due to the poor stability of TA and PT in an alkaline environment. Figure 8c shows the WCAs of treated membranes in NaCl solutions (0–5 wt%) were 0° and UOCAs ranged from 155° to 168.6°. Figure 8d illustrates the separating property of PVDF@TA/PT3 for petroleum ether emulsion separation in varying salt concentrations. The separation efficiencies were above 99.1%, and fluxes were above 150 L m^−2^ h^−1^. The findings indicated that the PVDF@TA/PT3 exhibited good stability in a salt environment.

**Figure 9:**
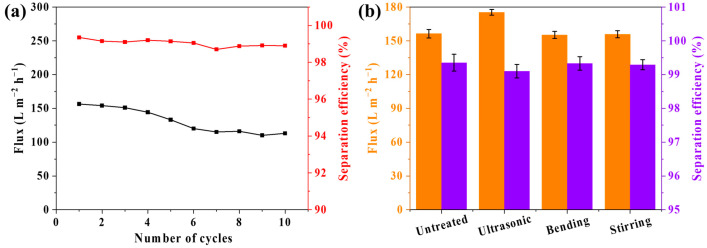
Separation performance of PVDF@TA/PT3 (**a**) for 10 cycles and (**b**) after treatment in different ways.

Membrane durability is an important consideration in emulsion separation applications. In this work, 10 consecutive oil–water separation cycles were performed to test the durability of the PVDF@TA/PT3 membrane. After each cycle, the membrane was rinsed with deionized water, and the next cycle started. Durability tests (Figure 9a) demonstrate that after 10 separation cycles, the membrane retains an efficiency of 99% and a flux of 120 L m^−2^ h^−1^. The separation performance of the membranes subjected to three independent treatments: (1) 60 times of bending and folding, (2) 12 h immersion in deionized water under stirring, and (3) 20 min ultrasonic treatment are comparatively presented in Figure 9b. Membranes subjected to bending and water immersion exhibit minimal changes in flux and efficiency. That only the membrane that had been treated with ultrasound showed significant changes in flux and efficiency. Ultrasonic treatment enhances membrane porosity, thereby resulting in improved permeation flux. The results illustrated that the PVDF@TA/PT3 exhibits favorable durability. The separation performance of modified membranes by different methods is summarized in Table 1.

**Figure 10:**
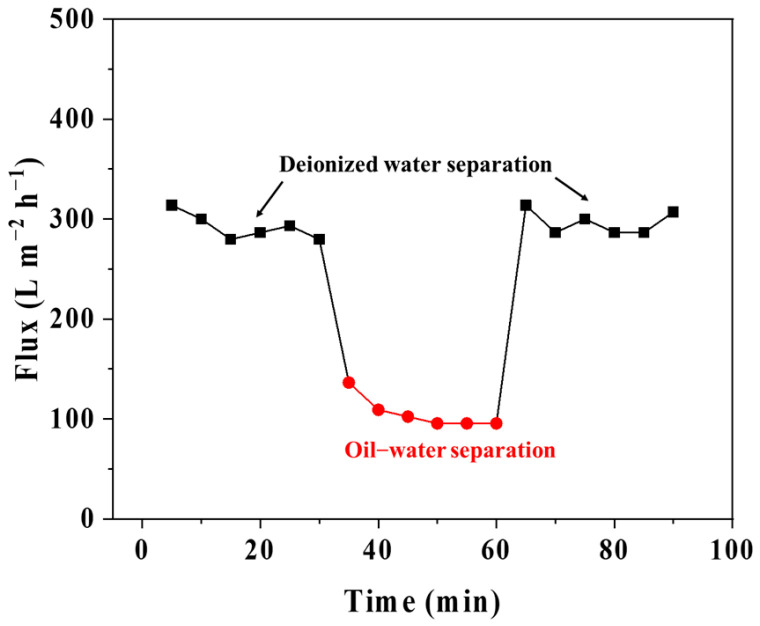
Time-dependent flux of the PVDF@TA/PT3 membrane.

The antifouling performance of the PVDF@TA/PT3 membrane was evaluated by three-step separation of water/oil–water emulsion/water. Figure 10 shows the flux of the PVDF@TA/PT3 membrane over time at different steps. The PVDF@TA/PT3 membrane exhibits stable permeation flux for pure water. When oil–water emulsion separation is carried out, oil drops are trapped by the membrane and deposited on the membrane surface or pore wall, so the separation flux of emulsion decreases significantly and gradually. After emulsion separation for 30 min, the membrane is cleaned thoroughly by deionized water. The water flux has almost returned to its initial level, mainly due to the excellent superwettability. The experimental results show that the membrane exhibits good antifouling performance.

### 3.5. Separation Mechanism

**Figure 11:**
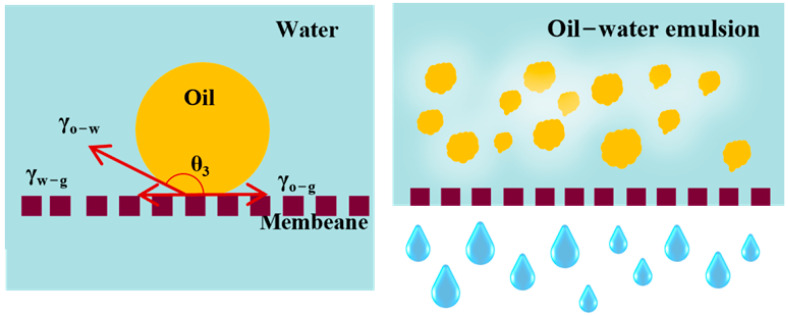
Mechanism of oil–water separation by PVDF@TA/PT membrane.

The separation mechanism of emulsion separation by PVDF@TA/PT is intricately tied to the synergistic effect of tannic acid (TA) and pectin (PT) on the membrane surface. Upon the sequential co-deposition of TA and PT onto the PVDF membrane, a hydrophilic layer is formed, significantly altering the membrane’s surface properties. As shown in Figure 11, the presence of TA and PT results in the formation of a hydrophilic layer on the surface of the PVDF membrane. Water molecules are the first to come into contact with the modified membrane surface and form a hydrated layer in emulsion separation, which acts as a barrier to prevent oil droplets from passing through the membrane. In the context of underwater superoleophobicity, Young’s equation can be formulated as follows:

(3) $$cos\theta_{3}=\frac{\gamma_{o-g}{cos\theta }_{1}-\gamma_{w-g}{cos\theta }_{2}}{\gamma_{o-w}}$$

where *θ*_3_ denotes the contact angle of oil droplets underwater, *γ_w−g_* represents the interfacial tension between water and gas, and *γ_o−g_* represents the interfacial tension between oil and gas. Given that the contact angles of both oil and water in air, *θ*_1_ and *θ*_2_, respectively, are zero for the PVDF@TA/PT membrane, and *cosθ*_1_ and *cosθ*_2_ both equal 1. Furthermore, considering the surface tensions of oil (ranging from 18.4 to 33.1 mN m^−1^) and water (72.8 mN m^−1^), the calculation based on Young’s equation reveals that cos*θ*_3_ is less than zero, indicating that *θ*_3_ must be greater than 90°.

This theoretical underpinning confirms the experimentally observed underwater superoleophobicity of the PVDF@TA/PT membrane, wherein oil droplets are repelled by the membrane surface while water molecules readily penetrate, enabling efficient oil–water separation. Consequently, the oil components are effectively blocked, whereas the aqueous phase freely passes through the membrane, achieving high separation performance in emulsion separation.

## 4. Conclusions

The PVDF@TA/PT membrane was synthesized by simple co-depositing of TA/PT on the commercially available PVDF membrane surface. The PVDF@TA/PT3 membrane has superhydrophilicity and underwater superoleophobicity, showing outstanding separation performance for various emulsions driven solely by gravity, maintaining 99% efficiency after 10 cycles, and demonstrating favorable durability in harsh environments. Moreover, the PVDF@TA/PT membrane exhibited favorable separation efficiency and flux after being treated by multiple methods. These findings demonstrate that the PVDF@TA/PT membrane maintained good durability in harsh environments. The modified membrane displays excellent performance and high stability, making it a promising candidate for industrial-scale oily wastewater treatment applications.

## Figures and Tables

**Table 1:** Flux and separation efficiency of different modified membranes.

Membranes	Flux (L m^−2^ h^−1^)	Separation Efficiency (%)
PVDF@TA3	143.1	99.05
PVDF@PT3	129.5	98.75
PVDF@TA/PT1	139.3	99.20
PVDF@TA/PT3	156.3	99.35
PVDF@TA/PT5	149.5	99.23
PVDF@TA/PT3 (1 wt%)	150.6	99.27
PVDF@TA/PT3 (2 wt%)	153.8	99.20
PVDF@TA/PT3 (3 wt%)	149.9	99.24
PVDF@TA/PT3 (4 wt%)	150.0	99.30
PVDF@TA/PT3 (5 wt%)	155.0	99.18
PVDF@TA/PT3 (pH = 1)	146.2	99.15
PVDF@TA/PT3 (pH = 3)	150.4	99.10
PVDF@TA/PT3 (pH = 5)	153.1	99.20
PVDF@TA/PT3 (pH = 9)	148.3	99.00
PVDF@TA/PT3 (pH = 11)	110.2	98.95
PVDF@TA/PT3 (pH = 13)	84.9	99.00

## Data Availability

The data that support the findings of this study are available within the article.

## Abbreviations

The following abbreviations are used in this manuscript:

TA	Tannic Acid
PT	Pectin
